# Physical and psychological (in)stability in extreme situations: physics models for understanding health stability

**DOI:** 10.1192/j.eurpsy.2022.1604

**Published:** 2022-09-01

**Authors:** F. Baessler, R. Willa

**Affiliations:** 1 Centre for Psychosocial Medicine, Department Of General Internal And Psychosomatic Medicine, Heidelberg, Germany; 2 Institute for Condensed Matter Theory, Karlsruhe Institute Of Technology, Karlsruhe, Germany

**Keywords:** psychology, Covid-19, mental healthcare, Susceptibility

## Abstract

**Introduction:**

The COVID-19 pandemic has shown how quickly and drastically everyday life can change in extreme situations.

**Objectives:**

To investigate how external factors can affect human health – mentally and physically – and what indicators herald the proximity to a critical upheaval.

**Methods:**

Using theories from theoretical physics and psychology, researchers from Heidelberg University and Karlsruhe Institute of Technology will observe emotional reaction via an ‘infinitesimal stimulus’ (f ) to an image that gives the ‘infinitesimal displacement’ (d). While both the stimulus and the reaction are chosen to be small – and hence keep a person well within their emotional stability – the ratio (d/f ) provides us a quantitative measure of the individual’s susceptibility i.e. reaction sensitivity. Over a six-month phase, we hope to correlate the individual susceptibility with the person’s general emotional state and to define a threshold reaction to indicate a person’s proximity to an emotional instability. Semi-structured interviews of extreme cases give us further insight into correlations between emotional states and susceptibility.

**Results:**

If an increased susceptibility in an individual actually precedes a long-term change in mood, then regular susceptibility measurements can be used, for instance, to detect depression at an early stage. We are particularly curious to observe the extent to which models from physics can be applied to society and the individual.

**Conclusions:**

The final output is to integrate practical implementation aspects into the medical curricula in a transdisciplinary manner. If possible, a formula for understanding health stability should be formulated that would be highly innovative for the medical field.

**Disclosure:**

This study is funded by the Heidelberg Academy for Sciences and Humanities.

